# Rapid Sequence Intubation in Traumatic Brain-injured Adults

**DOI:** 10.7759/cureus.2530

**Published:** 2018-04-25

**Authors:** Nicholas Kramer, David Lebowitz, Michael Walsh, Latha Ganti

**Affiliations:** 1 Emergency Medicine, University of Central Florida College of Medicine, Orlando, USA; 2 Office of Faculty and Academic Affairs, University of Central Florida College of Medicine, Orlando, USA; 3 Clinical Sciences, University of Central Florida College of Medicine, Orlando, USA

**Keywords:** rapid sequence intubation, traumatic brain injury (tbi), intubation, ketamine, emergency medicine, rocuronium, succinylcholine, pretreatment, induction agents, intracranial pressure

## Abstract

Deciding on proper medication administration for the traumatic brain injury (TBI) patient undergoing intubation can be daunting and confusing. Pretreatment with lidocaine and/or vecuronium is no longer recommended; however, high-dose fentanyl can be utilized to help blunt the sympathetic stimulation of intubation. Induction with etomidate is recommended; however, ketamine can be considered in the proper patient population, such as those with hypotension. Paralysis can be performed with either succinylcholine or rocuronium, with the caveat that rocuronium can lead to delays in proper neurological examinations due to prolonged paralysis. Recommendations for post-intubation continuous sedation medications include a combination propofol and fentanyl in the normotensive/hypertensive patient population. A combination midazolam and fentanyl or ketamine alone can be considered in the hypotensive patient.

## Introduction and background

Rapid sequence intubation (RSI) in the patient with traumatic brain injury (TBI) is a changing area of research. Airway control is essential for patients with TBI, as hypoxemia and hypercarbia lead to significant morbidity and mortality. However, we must take into consideration the fact that RSI also has the potential to worsen brain injury. The simple but essential act of laryngoscopy and placing of the endotracheal (ET) tube may stimulate the dense sympathetic and parasympathetic network traversing the pharynx and trachea. In addition to risks posed directly by the gag and cough reflex, sympathetic stimulation can cause an increased heart rate (HR), increased blood pressure (BP), and increased intracerebral pressure (ICP), while parasympathetic stimulation may trigger bronchospasm. It has been shown that the act of laryngoscopy alone increases systolic blood pressure (SBP) by a mean of 20 mmHg [1-2]. The effect on ICP has not been studied directly, but endotracheal suctioning has been shown to increase ICP by a minimum of 5 mmHg [3-4]. The increase in ICP from the sympathetic surge can cause an increase in cerebral blood volume, cerebral edema, and development of worsening hemorrhage or hematoma [5-6]. In addition, we must balance this against the risk of hypotension, as this can also increase both mortality and brain injury. Sedation carries many of the same concerns in addition to the needs to minimize anxiety, prevent agitation, allow manipulation of mechanical ventilation, and facilitate neurological assessments [7]. In this article, the authors explore the latest adult literature and summarize the data regarding agents for pretreatment, induction, paralysis, and sedation with the goal of preventing secondary brain injury.

## Review

Pretreatment

Lidocaine

Lidocaine has historically been used as a pretreatment in TBI as it was believed to lessen the sympathetic stimulation associated with RSI. However, evidence has been mixed. Two small studies have shown that lidocaine minimized an increase in ICP during neurosurgical procedures [8] or ET suctioning [9], while three studies showed no benefit during RSI [10-11] or ET suctioning [12]. Due to a lack of evidence in blunting ICP, weighed with the risk of potential side effects including hypotension [13], experts now recommend against using lidocaine for pretreatment in RSI for TBI [6, 14].

Defasciculating Dose of a Non-Depolarizing Agent

Pretreating with a low dose of a non-depolarizing agent, such as vecuronium, theoretically may blunt the rise in ICP due to muscle fasciculations when succinylcholine is used for RSI. There is strong evidence that succinylcholine increases ICP in patients undergoing neurosurgery for brain tumors (Marsh ML et al.: Succinylcholine-intracranial pressure effects in neurosurgical patients (Abstract). Anesth Analg 1980; 59: 550-1) and that a defasciculating dose of a non-depolarizing agent reduced increases in these select patients [15-16]. However, there are multiple small studies that suggest that succinylcholine does not increase ICP in head-injured patients and no studies that demonstrate that a non-depolarizing agent would affect ICP in these patients [17]. The current recommendation is against the use of a defasciculating dose of a non-depolarizing agent in TBI patients undergoing RSI with succinylcholine [14].

Fentanyl/Remifentanil

Several studies demonstrate that fentanyl attenuates rises in BP and HR in RSI [18-20]. By decreasing the cardiovascular response of sympathetic stimulation, fentanyl is thought to blunt increases in ICP related to laryngotracheal stimulation in RSI. One study by Kim et al. compared remifentanil versus lidocaine for attenuating the hemodynamic response during RSI. It found that lidocaine (at 1.5 mg/kg) had no effect, whereas remifentanil (at 1 mcg/kg) did blunt the hemodynamic response associated with RSI [21]. Fentanyl (at 2-3 mcg/kg) is currently recommended for neuroprotection in patients with increased ICP [14].

Induction   

Etomidate

Etomidate is well liked in the RSI world due to its mild hemodynamic profile [22-26]. This is particularly true during RSI in TBI, as a drop in mean arterial pressure (MAP) will cause a decrease in the cerebral perfusion pressure (CPP). In addition, etomidate has been shown to decrease cerebral blood flow and cerebral metabolic demand, all while preserving CPP [26]. One downside to this drug is that it has no analgesic properties; thus, neuroexcitation can be a concern if not properly mitigated [27].

Ketamine

Like etomidate, ketamine is exceptionally hemodynamically stable [28-29] but has the added benefit of possessing analgesic properties. Despite this, ketamine has been generally contraindicated for use in patients with TBI due to concerns of sympathetic stimulation, leading to an increase in the ICP. A study by Filanovsky et al. in 2010 examined the origins of this practice and determined that many of the studies that concluded that ketamine increased ICP were from the 1970s and were of questionable quality [30]. Additionally, ketamine may, in fact, be neuroprotective due to an increase in MAP and CPP [30-31], without increasing cerebral oxygen consumption or reducing regional glucose metabolism [32-33]. In a 2016 retrospective study of 968 adult trauma patients who underwent RSI with using either etomidate or ketamine, the authors found no difference in mortality or other patient-centered outcomes between the two induction agents [34]. In the 2011 Update from American College of Emergency Physicians (ACEP) Clinical Practice Guidelines for Ketamine Sedation, head trauma is no longer a relative contraindication, though ketamine remains relatively contraindicated for patients with central nervous system masses, abnormalities, or hydrocephalus [31]. Ketamine may best be suited for use in TBI patients with normal to low BP due to its potential for increasing the MAP and CPP [27].

Paralysis

Succinylcholine

Succinylcholine is a depolarizing neuromuscular blocking agent with rapid onset and offset properties. The rapid offset is beneficial as it allows for early neurological examinations. Concerns have been raised over the theory that muscle fasciculations could lead to increased ICP in these delicate patients; however, a 2001 study summarized the available literature on this subject and, though limited, concluded that the evidence does not support the hypothesis that succinylcholine causes an increase in ICP for head-injured patients [17].

Rocuronium

Strong opinions exist when comparing succinylcholine to rocuronium. While a large systematic review demonstrated superior intubating conditions when using succinylcholine [35], it is likely the agents are nearly identical in clinical practice [36]. In 2015, the Cochrane database investigators updated a previous review of RSI medications to include 11 additional studies to the previous 37 randomized controlled trials (RCT) and controlled clinical trials analyzed in 2008 [35]. Overall, the reviewers found succinylcholine was superior to rocuronium at achieving both acceptable intubating conditions and excellent intubating conditions when succinylcholine was dosed at least 1 mg/kg and rocuronium was dosed at least 0.6 mg/kg for RSI. However, further analysis revealed no statistical difference in intubation conditions when succinylcholine was compared to rocuronium dosed at 1.2 mg/kg.

While the majority of studies comparing succinylcholine to rocuronium have evaluated their effect on intubation conditions for RSI, intubation conditions may not necessarily translate into success of intubation or the number of intubation attempts required. In 2011, a retrospective study of all RSIs performed using succinylcholine or rocuronium was collected from a tertiary care emergency department over 15 months [37]. Of the total 327 RSIs performed, the rate of first attempt intubation success was similar between the succinylcholine and rocuronium groups (72.6% vs. 72.9%, p = 0.95). While the results of this study found the two paralytic agents to be equivalent with regard to first attempt intubation, the median dose of rocuronium used was 1.19 mg/kg (interquartile range (IQR) = 1 - 1.45 mg/ kg). The authors of the study concluded that higher doses of rocuronium may be necessary to achieve the equivalent effects of succinylcholine. A 2016 retrospective cohort study of 233 TBI patients requiring intubation in the emergency department was performed to help fill an important gap in the literature on the preferred paralytic for RSI in patients with TBI [38]. Patients either received succinylcholine or rocuronium to facilitate RSI. The two patient groups were similar and shared the same mortality rate of 23%. For patients with a low head Abbreviated Injury Scale (AIS) severity (AIS 0 to 3), there was no difference in mortality between the two groups. However, for patients with a high head AIS score (4 to 6), succinylcholine was associated with increased mortality compared with rocuronium (44% vs 23%, odds ratio (OR) 4.10, 95% confidence interval (CI) 1.18 - 14.12; p = 0.026). This was the first comparative study between succinylcholine and rocuronium to evaluate their effects on mortality in TBI patients. While succinylcholine use for RSI in patients with severe TBI was associated with increased mortality, it may not be possible to discriminate which patients are likely to benefit from avoiding succinylcholine at the time of presentation. Prospective clinical trials would help confirm these findings.

Due to its long duration of action, sustained paralysis with rocuronium can prevent repeat neurologic assessments. Patients receiving rocuronium have also been shown to receive less sedation and analgesia in the immediate post-intubation phase because the induced paralysis may make it appear as if they are calm and sedated [39]. A recent study published in 2015 found the average time to sedation in patients who received rocuronium for RSI was 55 minutes [39]. Considering the elimination half-life of etomidate, ketamine, and propofol are all less than 15 minutes, this reveals an alarmingly high incidence of patients paralyzed after RSI but without sedation. Additionally, care needs to be taken if opioids are used as remifentanil may delay the onset of paralysis by 30 - 45 seconds [40]. Currently, there is not enough data to recommend rocuronium over succinylcholine for RSI in TBI.

Post-intubation sedation/analgesia

Propofol

Propofol is advantageous over other medications in this category in that it as a rapid onset of action and short duration of action. This allows the effects to be rapidly eliminated from the patient, allowing for neurological examination, and then quickly titrated back to full effect. Care must be taken when selecting propofol in the hypotensive patient as it may lower the patient’s MAP, decreasing the body’s ability to maintain cerebral blood flow. Propofol’s cerebrovascular effects result in a reduction of episodes of intracranial hypertension (in the continuously monitored patient), and while there is some evidence that this agent may have neuroprotective effects in cases of mild TBI, this result has not been demonstrated in moderate to severe cases [41-42]. Finally, it must be noted that propofol has no analgesic effect, necessitating the use of additional medications for pain and comfort.

Midazolam

Midazolam boasts a relatively neutral hemodynamic profile, though some have raised concerns regarding its potential to lower systemic BP and thus, CPP [43]. Midazolam has a relatively fast onset and offset of action initially (half-life one hour), though tissue accumulation over time may result in delayed awakening. This effect may be the reason that midazolam has been associated with prolonged coma, increased ventilator days, and more ICU days when compared to propofol [7]. Midazolam’s additional benefits to the patient include its anxiolytic and anticonvulsant properties [41]. In a comparison study by Sandiumenge et al. [44], no significant difference was noted between midazolam and propofol in regard to a decrease in ICP and both agents demonstrated similar CPP. Analogous to propofol, midazolam also lacks analgesic properties and thus, is often paired with an opioid (such as fentanyl).

Fentanyl/Remifentanil

Fentanyl is a commonly utilized medication for analgesia after intubation, though it lacks appropriate sedation properties. As discussed above, attenuating pain may benefit the patient by minimizing the sympathetic response of elevated MAP and HR. While the hemodynamic properties of fentanyl are thought to be relatively neutral compared to other opioids, multiple studies have shown that bolus doses produce clinically significant increases in ICP, while decreasing MAP and CCP [45-46]. Thus, care must be taken to utilize the minimal appropriate dose for these patients. Fentanyl has a short duration of action, when given intravenously (IV), with the analgesic effect lasting approximately 30 - 60 minutes. Remifentanil is an ultra-short-acting opioid with analgesic effects lasting five to 10 minutes, which may allow for earlier neuro checks than fentanyl [47].

Ketamine

As discussed above, ketamine has historically been avoided in TBI patients due to the concern over raising the ICP. This dogma has been shown to be based on little, poorly conducted research from the 1970s. More recent studies have countered this data and shown that ketamine may have beneficial properties when utilized in the appropriate patient population [30-33, 48]. In a small study of eight TBI patients under propofol sedation, the addition of ketamine had no effect on HR, MAP, or CPP, while demonstrating the beneficial effect of lowering ICP [49]. Additionally, ketamine has the unique property of simultaneous sedation and analgesia, which may minimize the need for additional medications. Ketamine has a relatively long half-life at 2.5 hours, which limits the ability for neurological checks. As discussed in the Induction section, ketamine may best be utilized in TBI patients with normal to low BP as it may increase the patient’s MAP and CPP [27].

Discussion

RSI in the TBI patient is complex. The coordinated stages of pretreatment, induction, paralysis, sedation, and analgesia each present their own benefits and pitfalls. Much of this information is based on theoretical risks and benefits, as large randomized case-control studies looking at specific questions of interest with patient-centered outcomes are scarce, inadequate, or non-existent at this time.

The fundamental aim of our recommendations is to limit secondary brain injury due to RSI as measured by neurologic outcomes and mortality. When these indicators are not available, we focus on the effects on MAP, ICP, and CPP to the best of our current knowledge. A protocol for RSI in the TBI patient can be broken down into two arms: the hypotensive patients and the normotensive/hypertensive patients. We believe that the medication choices in these two groups should differ significantly as the cerebrovascular effects are diverse.

Pretreatment

The authors recommend no pretreatment medications in the hypotensive group. In the normotensive/hypertensive population, fentanyl IV bolus (2-3 mcg/kg) 3 minutes prior to induction is recommended. Fentanyl has been shown to blunt the sympathetic response of elevated MAP and HR during RSI. While fentanyl is relatively hemodynamically neutral, it does have the potential to decrease MAP and CCP when given at bolus doses [45-46]. This is why we recommend it as pretreatment only in the patients where hypotension is not a significant concern. Lidocaine and low-dose non-depolarizing agents are not recommended by current guidelines as the most recent evidence does not support their use.

Induction

For induction, the authors recommend ketamine in the hypotensive group and etomidate in the normotensive/hypertensive group. The previously held beliefs that ketamine was contraindicated in RSI have been successfully dispelled, and the most recent evidence suggests that it can be neuroprotective without increasing cerebral oxygen consumption or reducing regional glucose metabolism [30-31]. Ketamine may have sympathetic stimulation properties which lead to an increase in MAP and CPP; thus, the authors only recommend it in the hypotensive patient.

For the normotensive/hypertensive group, we believe the best induction medication is etomidate. Etomidate’s mild hemodynamic profile, along with evidence that it may decrease cerebral blood flow and cerebral metabolic demand while preserving CPP [26], makes it a strong candidate for this patient population.

Paralytic

The authors recommend succinylcholine as the paralytic agent of choice for both categories in our RSI protocol. Concerns regarding fasciculations and increased ICP with succinylcholine use have not been shown to be valid in the literature [17]. Succinylcholine’s rapid offset is exceptionally beneficial as it permits early neurological examinations. Rocuronium is gaining some traction for RSI in the TBI patient; however, with its prolonged duration of paralysis and limited data supporting its use over succinylcholine, we cannot yet recommend its use, except when succinylcholine is contraindicated.

Post-intubation Sedation/Analgesia

A systematic review from 2011 of many of the commonly used agents for sedation in TBI, including propofol, ketamine, etomidate, and agents from the opioid, benzodiazepine, α-2 agonist, and antipsychotic drug classes, concluded that no drug was superior in terms of neurological outcomes or mortality in the general traumatic brain injured patient [7]. Regardless, we believe that certain populations may benefit from one medication over another, though more research is certainly required.

In the hypotensive patient, we suggest a combination of midazolam and fentanyl, as this pairing has limited hemodynamic effects while simultaneously owning relatively short half-lives. Ketamine may be considered for this population as well, as its sympathetic effects may slightly elevate the MAP. However, ketamine has a relatively long duration of action which will limit neurological checks.

For the normotensive/hypertensive patient, our protocol calls for propofol and fentanyl. Propofol has the potential to lower the MAP further than other agents in this class, which may be beneficial in the hypertensive patient. Additionally, propofol may have neuroprotective effects in cases of mild TBI [42]. Fentanyl is added for comfort and may further benefit the hypertensive patient by blunting the sympathetic response to pain. Remifentanil may be substituted for fentanyl as the two medications have similar hemodynamic effects, with remifentanil allowing for a much more rapid offset of action for neurological checks.

Recommendations

**Figure 1:**
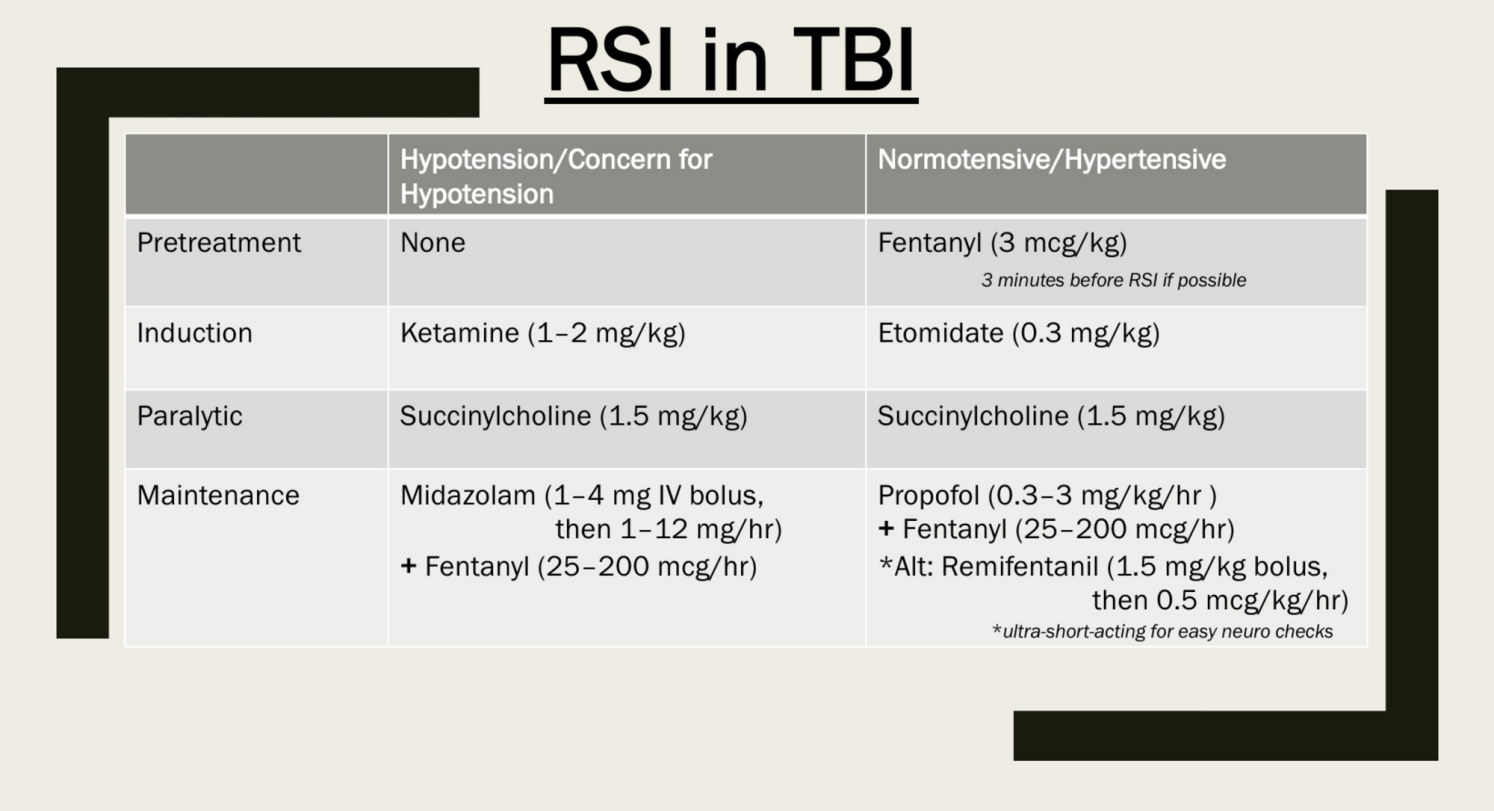
Summary Recommendations

Our recommendations, based on the above evidence, are summarized in Figure 1. 

## Conclusions

Medication administration for RSI in the patient with TBI is controversial and still not definitive. Patients with TBI are very sensitive to changes in hemodynamics; hence, adverse events can occur with improper medication administration. Further research and randomized control trials are needed to make formal recommendations.
